# The Role of Cholecystectomy in Hyperkinetic Gallbladder: A Retrospective Cohort Study in a Rural Hospital

**DOI:** 10.7759/cureus.29778

**Published:** 2022-09-30

**Authors:** Rachel Hart, Sri H Senapathi, Emma K Satchell, Shobha Mandal, Margaret McAndrew, Michael Scharf, Burt Cagir, Jean Miner

**Affiliations:** 1 Trauma and Acute Care Surgery, Guthrie Robert Packer Hospital, Sayre, USA; 2 General Surgery, Guthrie Robert Packer Hospital, Sayre, USA; 3 Internal Medicine, Guthrie Robert Packer Hospital, Sayre, USA; 4 Emergency Medicine, Geisinger Commonwealth School of Medicine, Sayre, USA; 5 General Surgery, Geisinger Commonwealth School of Medicine, Sayre, USA; 6 Colorectal Surgery, Guthrie Robert Packer Hospital, Sayre, USA

**Keywords:** biliary colic pain, minimally invasive cholecystectomy, cholecystectomy, biliary dyskinesia, hyperkinetic gallbladder

## Abstract

Background

Biliary dyskinesia is a functional gallbladder disorder in which there is an absence of a structural or mechanical cause for biliary pain. A cholecystokinin-hepatobiliary iminodiacetic acid (CCK-HIDA) scan is typically performed during workup, and cholecystectomy is the accepted treatment for low ejection fraction (EF) (less than 33%, as defined by the literature). However, few studies have examined the role of cholecystectomy in hyperkinetic gallbladder (EF ≥80%). The aim of our study was to examine symptom resolution following minimally invasive cholecystectomy in patients with hyperkinetic gallbladder.

Methodology

A retrospective chart review was conducted at Robert Packer Hospital in Sayre, PA. Patients who underwent minimally invasive cholecystectomy for biliary colic with EF ≥80% and who were without cholelithiasis on preoperative imaging or on final pathology were included in this study. The main outcome was symptom resolution at the postoperative visit. Data collected included age, gender, EF, body mass index, symptoms with CCK infusion, and pathology.

Results

A total of 48 patients were included. The mean age of patients was 41.2 years (standard deviation = 14.4), and the median age of patients was 42.2 years, with a range of 17-71 years. The majority of patients were female (83.3%). Overall, 58.3% of patients had replication of symptoms with CCK infusion. The mean gallbladder EF was 87.3%, with a median of 87.0 and a range of 80-98. In total, 33 (68.8%) patients had chronic cholecystitis on final pathology reports. There was a 95.9% symptom resolution rate among our patients two weeks postoperatively.

Conclusions

The overwhelming majority of patients experienced symptom resolution prior to their two-week postoperative visit following minimally invasive cholecystectomy for hyperkinetic gallbladder. These results strongly suggest a role of surgical management in patients with high EF.

## Introduction

Biliary dyskinesia is defined as a functional gallbladder disorder in the absence of a structural or mechanical cause for abdominal pain. It is diagnosed using the Rome IV criteria [1], which include biliary pain in the absence of structural pathology and support using a cholecystokinin-hepatobiliary iminodiacetic acid (CCK-HIDA) scan to measure ejection fraction (EF). Traditionally, an EF greater than 33% is considered normal, while patients with biliary dyskinesia have been classified by an EF less than 33% [2]. Cholecystectomy is considered the standard of care for low EF. Biliary dyskinesia is the indication for cholecystectomy in 10-20% of adults and is one of the most common indications for cholecystectomy in children, accounting for up to 50% performed in the pediatric population [3,4].

While cholecystectomy is a well-established treatment for low EF, there is less agreement regarding the role of cholecystectomy for those with hyperkinetic gallbladder, which is defined by an EF ≥80%. The purpose of this study was to evaluate symptom resolution in patients treated for hyperkinetic gallbladder with minimally invasive cholecystectomy at our institution. This article was previously presented as a podium abstract at the Society of American Gastrointestinal and Endoscopic Surgeons (SAGES) meeting in March 2022 [5].

## Materials and methods

A retrospective chart review was conducted to identify patients who underwent minimally invasive cholecystectomy for biliary colic at a rural tertiary care center, Robert Packer Hospital in Sayre, PA, USA, between January 2015 and March 2021. The Institutional Review Board of the Guthrie Clinic approved this study (approval number: 2102-08). The Rome IV criteria were used to identify patients. The Rome IV criteria are used for diagnosing a functional gallbladder disorder and are considered negative when biliary pain is present in the absence of gallstones or other structural pathologies [1]. We included all patients with biliary dyskinesia, a diagnosis of hyperkinetic gallbladder (EF ≥80% on CCK-HIDA scan), and laparoscopic or robotic cholecystectomy. We excluded patients with evidence of cholelithiasis on preoperative ultrasound or definitive cholelithiasis found on final pathology. Data collected from charts included age, gender, EF, body mass index (BMI), pathology, and presence of symptoms with CCK infusion during the HIDA scan. Because our hospital does not have a pediatric surgery section, all patients included were over 18 years of age. We reviewed charts for documented symptom resolution at the patient’s two-week postoperative visit, which was our primary outcome. Descriptive summary statistics were calculated.

## Results

A total of 48 patients were diagnosed with biliary colic, had high gallbladder ejection fraction (GBEF), and had no cholelithiasis found on ultrasound or final pathology following minimally invasive cholecystectomy (Table 1).

**Table 1:** Characteristics of patients and symptom resolution rate after cholecystectomy. SD: standard deviation; Min: minimum; Max: maximum; GBEF: gallbladder ejection fraction; CCK: cholecystokinin

Characteristics of patients	N = 48
Age	
Mean (SD)	41.2 (14.4) years
Median (Min, Max)	42.5 (17.0, 71.0)
Sex	
Female	40 (83.3%)
Male	8 (16.7%)
GBEF (%)	
Mean (SD)	87.3 (4.76)
Median (Min, Max)	87.0 (80.0, 98.0)
Chronic changes in gallbladder	
No chronic changes	15 (31.2%)
Chronic changes present	33 (68.8%)
Symptom replication with CCK	
Present	28 (58.3%)
Absent	5 (10.4%)
Unknown (not recorded)	15 (31.2%)
Symptom resolution after surgery	
Yes	46 (95.9%)
No	2 (4.2%)

**Figure 1:**
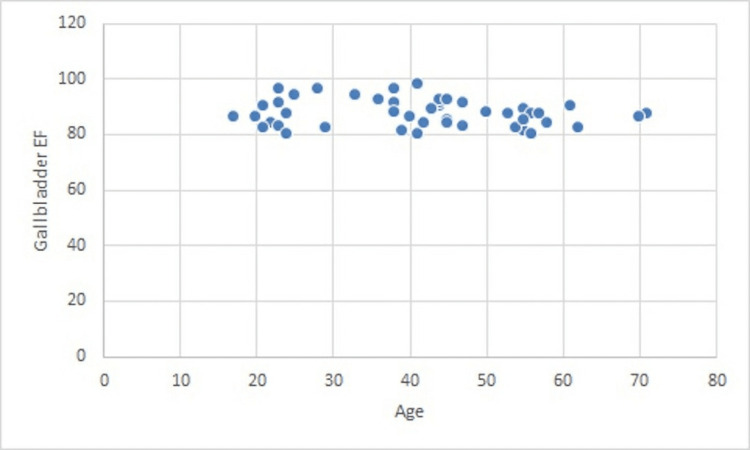
Gallbladder ejection fraction by age.

The mean age of the patients was 41.2 years (standard deviation = 14.4), and the median age of the patients was 42.2 years, with a range of 17-71 years. In total, 40 were female and eight were male patients. The average age of female patients was 40.0 years, while the average age of male patients was 47.4 years. GBEF was similar across ages (Figure 1).

The average BMI for all patients was 30.8 kg/m^2^. BMI was 31.06 for females kg/m^2^ and 29.5 kg/m^2^ for males. Overall, 58.3% of patients had a replication of symptoms with CCK infusion. The mean GBEF was 87.3% with a median of 87.0 and a range of 80-98. A total of 33 (68.8%) patients had chronic cholecystitis on their final pathology reports; the remaining 15 patients had unremarkable gallbladders. There was a 95.9% symptom resolution among these patients two weeks postoperatively.

## Discussion

Traditionally, patients who undergo HIDA scan in their workup for biliary disorders have been classified as either normal EF, defined as greater than 33%, or as low EF, defined as less than 33%. Biliary dyskinesia is associated with a low EF, and laparoscopic cholecystectomy is considered the gold standard of treatment.

Symptom resolution following cholecystectomy in patients with low EF has been well studied. A recent meta-analysis found that patients with a low EF were more likely to have symptom resolution following cholecystectomy than patients who were managed nonoperatively, with a relative risk of 2.37 [6]. A randomized controlled trial by Yap et al. [7] found that 91% of patients with a low EF had symptom resolution following surgery. One meta-analysis found that symptom resolution may be as high as 98% in those with biliary dyskinesia [8].

While cholecystectomy is the gold standard for low EF, its role is less clear for adults with an EF ≥80%, known as hyperkinetic gallbladder. In our study, 95.9% of patients with hyperkinetic gallbladder had symptom resolution following cholecystectomy. This is similar to the above-mentioned studies of symptom resolution in those with low EF, yet hyperkinetic gallbladder is not routinely considered for surgical management due to the traditional view that an EF above 33% is normal.

Symptom resolution in our study is similar to the few previous studies that have examined cholecystectomy for hyperkinetic gallbladder. A recent retrospective review by Whitaker et al. found a similar partial or complete symptom resolution rate of 92.8% [9]. An additional study reported cholecystectomy for hyperkinetic gallbladder resulted in partial or complete symptom resolution in 76% of patients [10]. A recent meta-analysis also found a similar symptomatic improvement of 91.3% [11]. One retrospective study compared those with hyperkinetic gallbladder who underwent surgery to those medically managed and found that those who underwent surgery had fewer emergency room visits, prescription medications, and diet modifications [12].

The pathophysiology behind biliary dyskinesia with high EFs is not well understood. One of the main hypotheses involves CCK, a hormone released by the I cells of the duodenum in response to fat and amino acids. It is thought that patients with hyperkinetic gallbladder have either increased CCK release or increased CCK receptors, causing excessive gallbladder contraction and subsequent abdominal pain. In our cohort, we noted that 58.3% of patients reported symptoms with CCK infusion, which may support this hypothesis.

The CCK hypothesis also suggests that rapid contraction of the gallbladder may cause inflammatory changes secondary to increased pressure [13]. Overall, 68.8% of patients in our study had chronic cholecystitis on histological examination. This is lower than the 80-95% of patients with hyperkinetic gallbladders reported to have chronic cholecystitis in other studies [11,13,14].

This is one of the largest single-center studies to examine hyperkinetic gallbladder. One of the main limitations of this study is its retrospective nature, which limited us to the information available within our electronic records. While we had a follow-up rate of 100% at two weeks, and all patients were questioned about symptom resolution, other data points were not always reliably documented. The presence or absence of symptoms with CCK infusion was not recorded in 31.2% of patients, although the majority of those documented did have symptom replication. This is an important consideration for determining if those who develop symptoms with CCK infusion will have symptom resolution after cholecystectomy. One area of future study may be the use of CCK infusion as a prognostic tool for symptom resolution after cholecystectomy in the preoperative evaluation of patients with hyperkinetic gallbladder. Future studies may additionally wish to follow patients longer-term to ensure permanent resolution of symptoms.

## Conclusions

We demonstrate that most patients with hyperkinetic gallbladder (96.9%) have symptom resolution following minimally invasive cholecystectomy. This suggests that surgical management should be strongly considered in the management of patients with symptoms of biliary colic and high EF and implies that this condition should be treated similarly to low EF.
